# Glaucocalyxin B Attenuates Ovarian Cancer Cell Growth and Cisplatin Resistance In Vitro via Activating Oxidative Stress

**DOI:** 10.1155/2022/6324292

**Published:** 2022-02-25

**Authors:** Tingting Zhang, Chenxin Xu, Peisen Zheng, Xiaoxian Zhang, Chenyu Qiu, Fengjiao Wu, Jundixia Chen, Zhongxiang Xiao, Jiandong Zhu, Jingjing Zhang, Peng Zou, Daoyong Ni

**Affiliations:** ^1^Affiliated Yueqing Hospital, Wenzhou Medical University, Wenzhou 325035, China; ^2^School of Pharmaceutical Sciences, Wenzhou Medical University, Wenzhou 325035, China; ^3^School of Nursing, Wenzhou Medical University, Wenzhou 325035, China

## Abstract

Ovarian cancer is one of the fatal gynecological cancers around the world. Cisplatin is the first-line chemotherapy drug for the clinical treatment of ovarian cancer. However, many patients with ovarian cancer are still suffering from resistance to cisplatin. Therefore, the new drug combinations or treatment strategies for ovarian cancer are urgently needed. Glaucocalyxin B (GLB), a diterpenoid isolated from the aerial parts of *Rabdosia japonica*, has shown antitumor activity in some tumors. However, the mechanisms by which GLB inhibits ovarian cancer remain unclear. In the present study, we showed that GLB potently inhibits ovarian cancer cell growth in a dose-dependent manner. Furthermore, we found that GLB has a notably synergistic antitumor effect with cisplatin. Mechanistically, we found that GLB enhances the sensitivity of ovarian cancer cells to cisplatin via increasing reactive oxygen species (ROS) levels, the phosphorylation of c-Jun N-terminal kinase (JNK), and DNA damage. Interestingly, a synergistic inhibitory effect of GLB with cisplatin was also observed in the cells which were resistance to cisplatin. Together, these data suggest that GLB can sensitize ovarian cancer cells to cisplatin by increasing ROS levels.

## 1. Introduction

Ovarian cancer is one of the leading causes of death from gynecological malignancies around the world. Due to the lack of specific signs or symptoms, ovarian cancer is particularly difficult to diagnose at an early stage [1]. The current standard treatment for ovarian cancer is chemoradiotherapy combined with surgical resection. However, survival figures of patients with ovarian cancer have not significantly improved over the recent decades [2]. The resistance to chemotherapy in ovarian cancer patients is a major cause of poor prognosis and high mortality [3, 4].

Cisplatin is a first-line chemotherapy drug against advanced ovarian cancer [5]. The cytotoxicity of cisplatin is mainly because of its interaction with DNA, which hinder RNA transcription and DNA replication and contributing to cell cycle arrest and apoptosis. Most patients with ovarian cancer are responsive to cisplatin. However, intrinsic or acquired resistance to cisplatin inevitably occurs [6, 7]. Therefore, the new drug combinations or treatment strategies for ovarian cancer patients are urgently needed [8].

Natural products are critical sources of lead molecules in anticancer drug discovery. Many anticancer drugs in clinical use are natural products or their derivatives [9, 10]. Glaucocalyxin B (GLB) is a diterpenoid isolated from the Chinese traditional medicine *Rabdosia japonica*, which has been known to possess anti-inflammatory and anticancer effects [11, 12]. It has been reported that cervical cancer and gastric cancer could be inhibited by GLB, but the effect of GLB in ovarian cancer remains unknown [12, 13]. Here, we showed that GLB caused DNA damage via increasing the levels of intracellular ROS and the phosphorylation of JNK. More importantly, we found that GLB could enhance the sensitivity of ovarian cancer cells to cisplatin. Overall, our study provides a rationale for evaluating the combination of GLB and cisplatin in the treatment of ovarian cancer patients.

## 2. Methods

### 2.1. Chemicals and Antibodies

Glaucocalyxin B (GLB) was obtained from Chengdu Herbpurify (Chengdu, China). Cisplatin and SP600125 were purchased from TargetMol (Boston, USA). N-acetyl-L-cysteine (NAC) was purchased from Aladdin Industrial Corporation (Shanghai, China). Crystal violet staining solution and 2,7-dichlorodihydrofluorescein diacetate (DCFH-DA) were purchased from Beyotime Biotechnology (Shanghai, China). Antibodies against phospho-SAPK/JNK (4668, 1 : 1000) and SAPK/JNK (9252, 1 : 1000) were purchased from Cell Signaling Technology (Danvers, USA). Antibody against GAPDH (10494-1-AP, 1 : 10000) was purchased from Proteintech Group (Wuhan, China). Antibody against 53BP1 (NB100-304, 1 : 2000) was obtained from Novus Biologicals (Littleton, USA).

### 2.2. Cell Lines and Cell Culture

The human ovarian cancer cell line A2780 was obtained from the Cell Resource Center of Peking Union Medical College. A2780/DDP cell line was generated by exposing A2780 cells to stepwise increasing concentrations of cisplatin. A2780 and A2780/DDP cells were cultured in Dulbecco's modified Eagle's medium (DMEM) with 10% FBS at 37°C in a humidified atmosphere containing 5% CO_2_. NRK-52E cells were cultured in DMEM with 10%FBS at 37°C in a humidified atmosphere containing 5% CO_2_. MIHA cells were cultured in RPMI-1640 medium with 10% FBS at 37°C in a humidified atmosphere containing 5% CO_2_.

### 2.3. Cell Viability

Cells were seeded in 6-well plates and allowed to adhere overnight in the incubator at 37°C.The cells were then treated with different concentrations of GLB or cisplatin for 24 h, and cell viability was determined by trypan blue exclusion. The percentage of viable cells relative to DMSO-treated cells is indicated. Combination index (CI) values were calculated using the Chou-Talalay method [14].

### 2.4. Western Blot Analysis

After various treatments, the cells were lysed in cell lysis buffer. Equal amounts of protein from each sample were loaded onto 10% SDS-PAGE gel and then transferred to PVDF membranes. The membranes were blocked with 5% skim milk in TBST for 2 h at room temperature. Then, the membranes were incubated with primary antibodies overnight at 4°C. HRP-conjugated secondary antibodies and ECL substrate were used for detection. The signal intensities were quantified using ImageJ software.

### 2.5. Measurement of Reactive Oxygen Species (ROS)

A2780 or A2780/DDP cells were seeded into 6-wellplates. After various treatments, the cells were stained with DCFH-DA probe (1 : 1000 dilution) at 37°C for 30 min based on the protocol of the ROS assay kit. A fluorescence microscope was used to detect the ROS levels.

### 2.6. Immunofluorescence Staining

A2780 or A2780/DDP cells were seeded on sterile cover glasses and treated with the test compounds for 20 h. For immunofluorescence, the cells were incubated with primary antibody 53BP1 overnight at 4°C and then incubated with the DyLight 488 conjugated secondary antibody for 1.5 h at room temperature. Next, the cells were washed and mounted with DAPI stain. Images were detected and captured with a fluorescence microscope.

### 2.7. Statistical Analysis

Data were analyzed using GraphPad Prism 5.0 software. One-way ANOVA with Bonferroni's post hoc test was used to determine the significance of the differences. *p* < 0.05 was considered statistically significant. *p* values < 0.05, < 0.01, and < 0.001 are indicated with one, two, and three asterisks, respectively.

## 3. Results

### 3.1. GLB Increases the ROS Levels and Inhibits Cell Growth in Human Ovarian Cancer Cells

**Figure 1:**
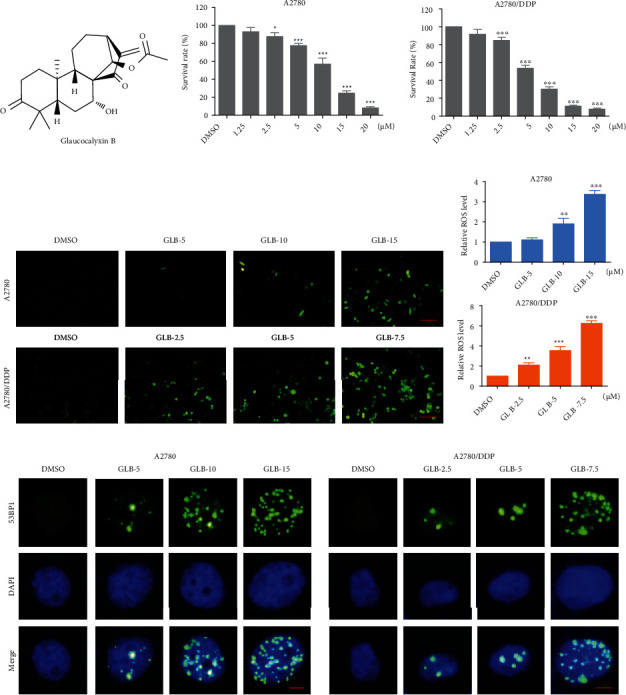
GLB increases the ROS levels and inhibits cell growth in human ovarian cancer cells. (a) The chemical formula of GLB. (b, c) A2780 or A2780/DDP cells were treated with various concentrations of GLB for 24 h. Cell viability was determined by trypan blue exclusion. (d, e) A2780 or A2780/DDP cells were exposed to GLB for 2 h; intracellular ROS levels were monitored by fluorescent probe DCFH-DA. Scale bar = 100 *μ*m. (f) A2780 or A2780/DDP cells were treated with GLB for 20 h; the formation of 53BP1 foci was analyzed using a fluorescence microscope. Scale bar = 5 *μ*m. Data from three technical replicates (^∗^*p* < 0.05, ^∗∗^*p* < 0.01, and ^∗∗∗^*p* < 0.001).

To identify the cytotoxicity of GLB (Figure 1(a)), A2780 and A2780/DDP cell lines were used in our experiments. As shown in Figures 1(b) and 1(c), GLB inhibited the growth of A2780 and A2780/DDP cells in a dose-dependent manner. Interestingly, GLB is more effective in A2780/DDP cells which were resistant to cisplatin. By contrast, GLB treatment has little effect on normal NRK-52E and MIHA cells (Fig. S1A-B). We then detected ROS accumulation in ovarian cancer cells. The result revealed that GLB significantly increased the ROS levels in A2780 and A2780/DDP cells (Figures 1(d) and 1(e)). Excessive accumulation of ROS can cause damage to DNA. As shown in Figure 1(f), GLB treatment caused an accumulation of nuclear 53BP1 foci in A2780 and A2780/DDP cells.

### 3.2. ROS Scavenger Prevents the GLB-Induced DNA Damage and Proliferation Inhibition in Human Ovarian Cancer Cells

**Figure 2:**
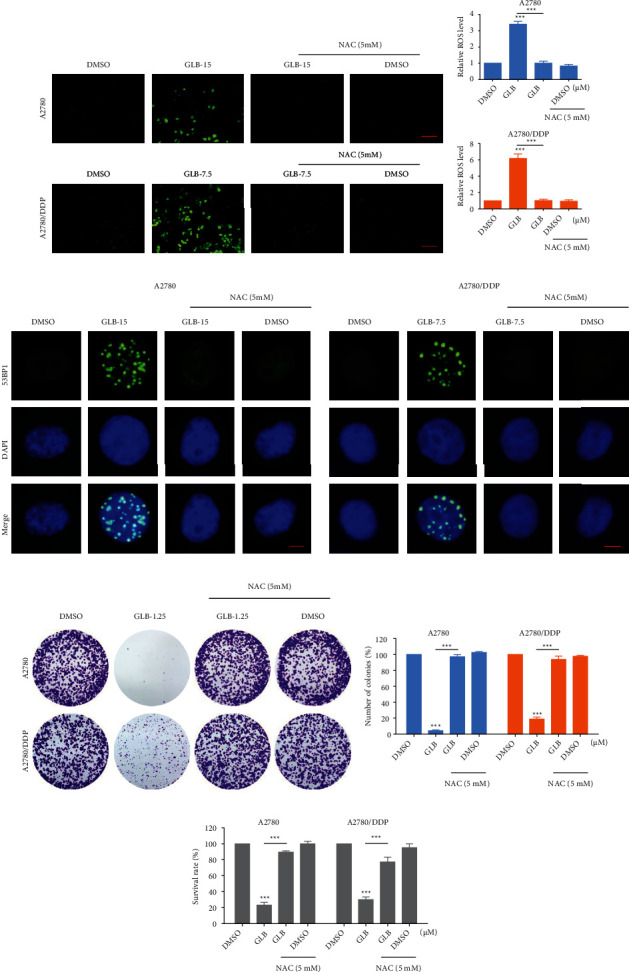
ROS scavenger prevents the GLB-induced DNA damage and proliferation inhibition. (a, b) A2780 or A2780/DDP cells were pretreated with ROS scavenger NAC (5 mM) for 1 h and then treated with GLB for 2 h; the intracellular ROS levels were monitored by fluorescent probe DCFH-DA. Scale bar = 100 *μ*m. (c) A2780 or A2780/DDP cells were pretreated with NAC for 1 h and then treated with GLB for 20 h; the formation of 53BP1 foci was analyzed using a fluorescence microscope. Scale bar = 5 *μ*m. (d, e) A2780 or A2780/DDP cells were pretreated with NAC for 1 h; the colony formation ability was measured by colony-forming assay after treatment with GLB. (f) A2780 or A2780/DDP cells were pretreated with NAC for 1 h and then treated with GLB for 24 h; the cell viability was determined by trypan blue exclusion. Data from three technical replicates (^∗∗∗^*p* < 0.001).

To determine whether ROS was involved in GLB-induced cell death, we pretreated A2780 and A2780/DDP cells with an antioxidant N-acetyl-L-cysteine (NAC) for 1 hour and found that NAC significantly reversed GLB-induced ROS accumulation (Figures 2(a) and 2(b)). In addition, the accumulation of nuclear 53BP1 was also blocked by NAC pretreatment (Figure 2(c)). Importantly, we found that pretreatment with NAC markedly reversed the GLB-mediated inhibitory effects on colony formation and cell viability in A2780 and A2780/DDP cells (Figures 2(d)–2(f)).

### 3.3. JNK Signaling Pathway Is Involved in GLB-Induced Cell Death

**Figure 3:**
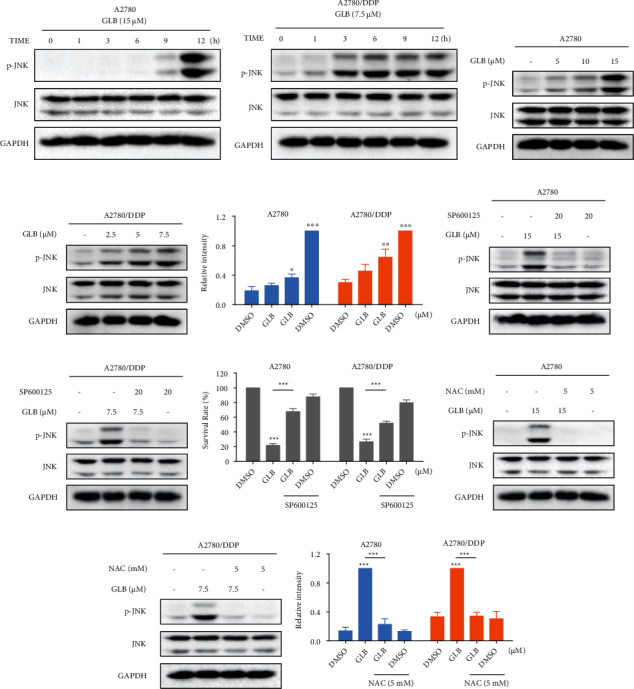
JNK signaling pathway is involved in GLB-induced cell death. (a, b) A2780 or A2780/DDP cells were treated with GLB at different time points. The protein levels of p-JNK were detected by western blot. (c–e) A2780 or A2780/DDP cells were treated with GLB at different concentrations. The protein levels of p-JNK were detected by western blot. (f, g) A2780 or A2780/DDP cells were pretreated with JNK inhibitor SP600125 (20 *μ*M) for 1 h before exposing to GLB; the protein levels of p-JNK were detected by western blot. (h) A2780 or A2780/DDP cells were pretreated with JNK inhibitor SP600125 (20 *μ*M) for 1 h before exposing to GLB; the cell viability was determined by trypan blue exclusion. (i–k) A2780 or A2780/DDP cells were pretreated with NAC for 1 h before exposing to GLB; the protein levels of p-JNK were detected by western blot. Data from three technical replicates (^∗^*p* < 0.05, ^∗∗^*p* < 0.01, and ^∗∗∗^*p* < 0.001).

We then assessed the level of JNK phosphorylation in human ovarian cancer cells. Time-course results showed that GLB treatment significantly increased the phosphorylation of JNK in A2780 and A2780/DDP cells (Figures 3(a) and 3(b)). Furthermore, GLB dose dependently increased the level of JNK phosphorylation (Figures 3(c)–3(e)). To determine whether the JNK signaling pathway was involved in GLB-induced cell death, the cells were treated with GLB after pretreated with SP600125, a JNK inhibitor. Western blot analysis showed that the phosphorylation of JNK induced by GLB was significantly blocked by SP600125 (Figures 3(f) and 3(g)). This was associated with a noticeable reduction in GLB-induced cell death in A2780 and A2780/DDP cells (Figure 3(h)), suggesting that the JNK signaling pathway is essential for the antitumor effect of GLB. Importantly, the increased phosphorylation of JNK was greatly reversed by NAC (Figures 3(i)–3(k)), indicating that the JNK signaling pathway is a downstream effector of ROS.

### 3.4. GLB and Cisplatin Synergize to Induce Cell Death in Human Ovarian Cancer Cells

**Figure 4:**
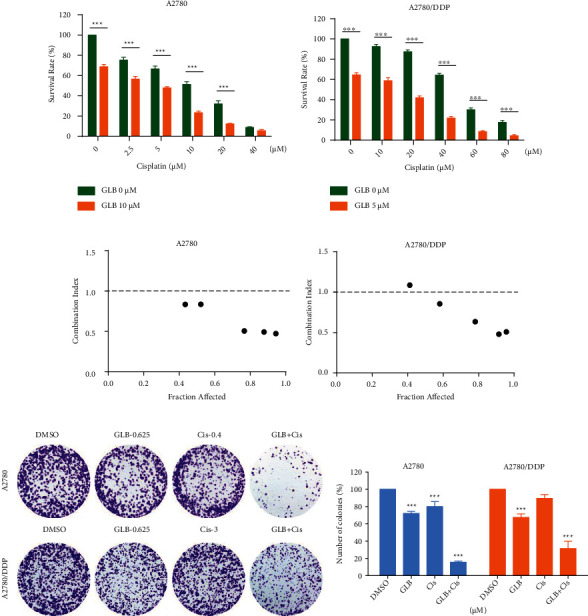
GLB as a potential sensitizer for cisplatin in human ovarian cancer cells. (a, b) A2780 or A2780/DDP cells were treated with GLB or cisplatin alone or their combination for 24 h; the cell viability was determined by trypan blue exclusion. (c, d) Combination index (CI) values were calculated using the Chou-Talalay method. (e, f) A2780 or A2780/DDP cells were treated with GLB or cisplatin alone or their combination; the colony formation ability was measured by colony-forming assay. Data from three technical replicates (^∗∗∗^*p* < 0.001).

To facilitate clinical translation, we then investigated the combined effects of GLB and cisplatin on the viability of A2780 and A2780/DDP cells. As shown in Figures 4(a) and 4(b), cisplatin inhibited the growth of A2780 and A2780/DDP cells as monotherapy, but the inhibitory effect was stronger when in combination with GLB. The combination index (CI) values suggested that the combination of GLB and cisplatin exhibited a significant synergistic effect in inhibiting ovarian cancer cels proliferation (Figures 4(c) and 4(d)). Further evidences were acquired from the colony-forming assay. Compared with GLB or cisplatin treatment alone, GLB combined with cisplatin significantly reduced the number of colonies formed in A2780 and A2780/DDP cells (Figures 4(e) and 4(f)).

### 3.5. GLB and Cisplatin Cooperate to Induce ROS-Mediated Cell Death in Human Ovarian Cancer Cells

**Figure 5:**
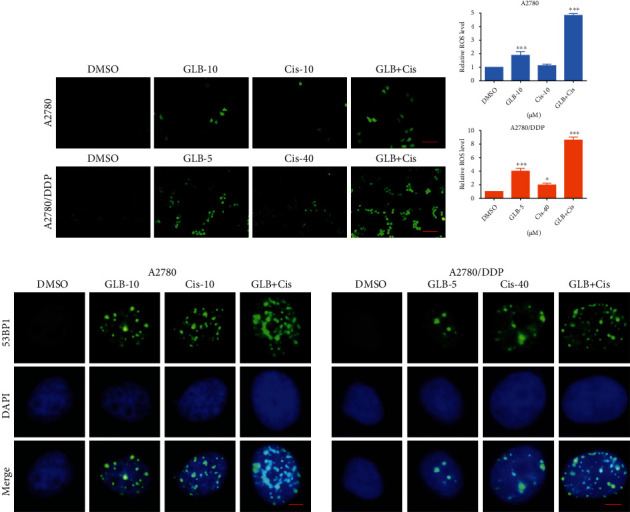
GLB and cisplatin cooperate to increase ROS levels. (a, b) A2780 or A2780/DDP cells were treated with GLB or cisplatin alone or their combination for 2 h; the intracellular ROS levels were monitored by fluorescent probe DCFH-DA. Scale bar = 100 *μ*m. (c) A2780 or A2780/DDP cells were treated with GLB or cisplatin alone or their combination for 20 h; the formation of 53BP1 foci was analyzed using a fluorescence microscope. Scale bar = 5 *μ*m. Data from three technical replicates (^∗^*p* < 0.05, ^∗∗∗^*p* < 0.001).

**Figure 6:**
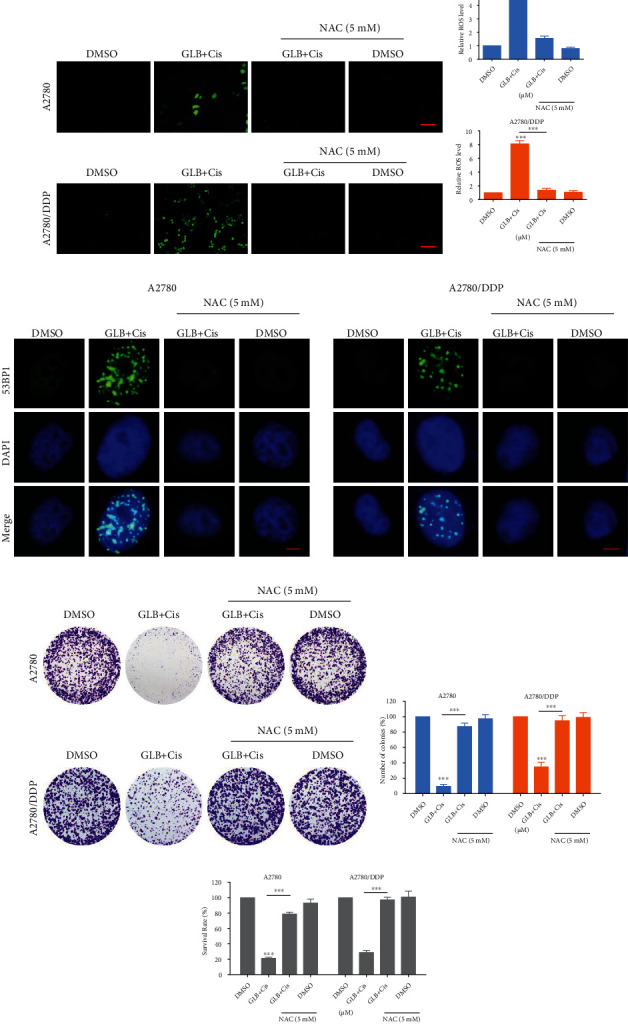
NAC pretreatment reversed the combined treatment-induced DNA damage and proliferation inhibition. (a, b) A2780 or A2780/DDP cells were pretreated with NAC for 1 h and then treated with GLB or cisplatin alone or their combination for 2 h; the intracellular ROS levels were monitored by fluorescent probe DCFH-DA. Scale bar = 100 *μ*m. (c) A2780 or A2780/DDP cells were pretreated with NAC for 1 h and then treated with GLB or cisplatin alone or their combination for 20 h; the formation of 53BP1 foci was analyzed using a fluorescence microscope. Scale bar = 5 *μ*m. (d, e) A2780 or A2780/DDP cells were pretreated with NAC for 1 h; the colony formation ability was measured by colony-forming assay after treated with the combination. (f) A2780 or A2780/DDP cells were pretreated with NAC for 1 h and then treated with the combination for 24 h; the cell viability was determined by trypan blue exclusion. Data from three technical replicates (^∗∗∗^*p* < 0.001).

To investigate the molecular mechanisms underlying the synergistic action of GLB and cisplatin, we detected ROS levels in ovarian cancer cells. As shown in Figures 5(a) and 5(b), compared with GLB or cisplatin treatment alone, GLB combined with cisplatin significantly increased the ROS levels in A2780 and A2780/DDP cells. Moreover, the accumulation of nuclear 53BP1 foci was significantly increased in A2780 and A2780/DDP cells after the combined treatment (Figure 5(c)). To determine whether ROS accumulation was involved in the synergistic action of GLB and cisplatin, we pretreated A2780 and A2780/DDP cells with NAC for 1 hour and found that NAC significantly reversed the combined treatment-induced ROS accumulation (Figures 6(a) and 6(b)). Moreover, the accumulation of nuclear 53BP1 was also blocked by NAC pretreatment (Figure 6(c)). Notably, we found that pretreatment with NAC markedly reversed the combined treatment-induced growth inhibition in A2780 and A2780/DDP cells (Figures 6(d)–6(f)), suggesting that ROS accumulation plays a critical role in the synergistic action of GLB and cisplatin.

### 3.6. GLB in Combination with Cisplatin Activate ROS-Mediated JNK Signaling Pathway in Human Ovarian Cancer Cells

**Figure 7:**
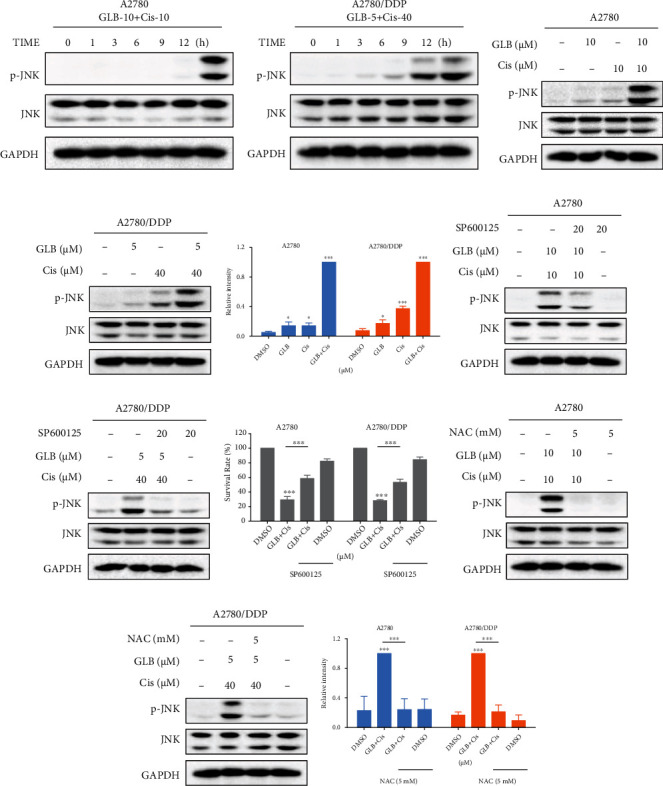
JNK signaling pathway is involved in the combined treatment-induced cell death. (a, b) A2780 or A2780/DDP cells were treated with GLB and cisplatin combination at different time points. The protein levels of p-JNK were detected by western blot. (c–e) A2780 or A2780/DDP cells were treated with GLB or cisplatin alone or their combination; the protein levels of p-JNK were detected by western blot. (f, g) A2780 or A2780/DDP cells were pretreated with SP600125 for 1 h before exposing to GLB and cisplatin combination; the protein levels of p-JNK were detected by western blot. (h) A2780 or A2780/DDP cells were pretreated with SP600125 for 1 h before exposing to GLB and cisplatin combination; the cell viability was determined by trypan blue exclusion. (i–k) A2780 or A2780/DDP cells were pretreated with NAC for 1 h before exposing to GLB and cisplatin combination; the protein levels of p-JNK were detected by western blot. Data from three technical replicates (^∗^*p* < 0.05, ^∗∗∗^*p* < 0.001).

We further investigated whether the JNK signaling pathway was involved in the synergistic action of GLB and cisplatin. Time-course results showed that GLB combined with cisplatin significantly increased the phosphorylation of JNK in A2780 and A2780/DDP cells (Figures 7(a) and 7(b)). Moreover, compared with GLB or cisplatin treatment alone, the phosphorylation of JNK was increased more significantly in the combination group (Figures 7(c)–7(e)). Further analysis showed that the phosphorylation of JNK induced by the combination of GLB and cisplatin was significantly blocked by SP600125 pretreatment (Figures 7(f) and 7(g)). This was associated with a noticeable reduction in the combined treatment-induced cell death in A2780 and A2780/DDP cells (Figure 7(h)), suggesting that the JNK signaling pathway is essential for the synergistic action of GLB and cisplatin. Importantly, the increased phosphorylation of JNK was greatly reversed by NAC pretreatment (Figures 7(i)–7(k)).

## 4. Discussion

**Figure 8:**
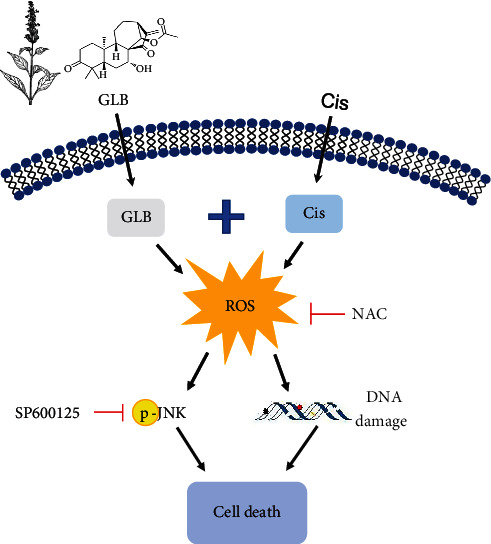
The proposed working model.

Despite the great advances in the treatment of ovarian cancer, the 5-year survival rates are still around 40%. One of the primary reasons for poor prognosis of patients with ovarian cancer is chemoresistance [1]. Recently, more attention has been concentrated on the combination therapy which could sensitize the ovarian cancer cells to conventional chemotherapy agents [15, 16]. Hence, novel drugs which are nontoxic and effective and can remarkably strengthen the effects of chemotherapy agents are urgently needed. In this study, we investigated whether GLB could enhance the antitumor effects of cisplatin in human ovarian cancer cells. The results showed that GLB has a notably synergistic antitumor effect with cisplatin in A2780 and A2780/DDP cells. Mechanistically, we found that GLB enhanced the sensitivity of ovarian cancer cells to cisplatin via the induction of ROS (Figure 8).

ROS are byproducts of cellular metabolic processes; they play an important role in regulating physiological processes, but excessive ROS production often leads to cellular senescence and apoptosis [17, 18]. Cancer cells display abnormal redox homeostasis, and hyperproliferation of cancer cells is accompanied by high ROS production. Elevated ROS production renders cancer cells more vulnerable to exogenous ROS-generating agents. Therefore, ROS-related therapeutic strategies are developed for cancer treatment [19–21]. In this study, we found that GLB combined with cisplatin significantly increased ROS levels in A2780 and A2780/DDP cells. Moreover, the cell death induced by the combination treatment was markedly blocked by NAC pretreatment, suggesting that ROS accumulation plays an important role in the synergistic action of GLB and cisplatin. These findings further support the notion that increasing ROS accumulation is a promising therapeutic strategy for cancer [22, 23].

Another interesting finding in the present study was that the JNK signaling pathway was markedly activated in response to the combination of GLB and cisplatin. Further analysis showed that pretreatment with SP600125 partially reversed the combined treatment-induced growth inhibition in A2780 and A2780/DDP cells, suggesting that the JNK signaling pathway is essential for the synergistic action of GLB and cisplatin. ROS has been reported to activate the JNK pathway leading us to speculate that ROS may be the upstream mediator of the JNK signaling pathway [24, 25]. Indeed, we found that the increased phosphorylation of JNK was greatly reversed by NAC pretreatment. These findings indicate that the JNK signaling pathway is a downstream effector of ROS, but the precise molecular mechanism underlying this relationship remains unclear. In recent years, network pharmacology technology has played a critical role in identifying effective ingredients, targets, and pharmacological mechanisms of traditional Chinese medicine [26, 27]. Further studies based on network pharmacology technology will be required to understand the relationship between ROS generation and activation of the JNK signaling pathway in the combined treatment-induced cancer cell death.

In conclusion, our study revealed the synergistic mechanism of GLB and cisplatin and demonstrated that the ROS-mediated JNK pathway was a crucial mediator for the antitumor action of GLB in combination with cisplatin in ovarian cancer cells. Our results suggest that GLB in combination with cisplatin may be a more effective therapeutic strategy for ovarian cancer. Further studies on the antitumor action and toxicity of the combination therapy with GLB and cisplatin *in vivo* are urgently needed.

## Data Availability

All datasets presented in this study are included in the article material.

## Supplementary Materials

Supplementary Materials Figure S1: GLB treatment has little effect on normal cells. (A, B) Cell viability was measured after treated with GLB for 24 h. Data from three technical replicates.
