# Supplementary material for: Glaucocalyxin B Attenuates Ovarian Cancer Cell Growth and Cisplatin Resistance In Vitro via Activating Oxidative Stress
Source: Oxid Med Cell Longev. 2022 Feb 25;2022:6324292. doi: 10.1155/2022/6324292 (PMC8896941; doi:10.1155/2022/6324292)
Supplement: Supplementary Materials — Figure S1: GLB treatment has little effect on normal cells. (A, B) Cell viability was measured after treated with GLB for 24 h. Data from three technical replicates. [file 6324292.f1.docx]

**Glaucocalyxin B attenuates ovarian cancer cell growth and cisplatin resistance in vitro via activating oxidative stress**

Tingting Zhang^1,2#^, Chenxin Xu^2#^, Peisen Zheng^2#^, Xiaoxian Zhang^3#^, Chenyu Qiu^2^, Fengjiao Wu^2^, Jundixia Chen^1^, Zhongxiang Xiao^1^, Jiandong Zhu^1^, Jingjing Zhang^3^, Peng Zou^1,2^*, Daoyong Ni^1^*

^1^ Affiliated Yueqing Hospital, Wenzhou Medical University, Wenzhou 325035, China

^2^ School of Pharmaceutical Sciences, Wenzhou Medical University, Wenzhou 325035, China

^3^ School of Nursing, Wenzhou Medical University, Wenzhou 325035, China

* Corresponding author: Peng Zou; Address: School of Pharmaceutical Sciences, Wenzhou Medical University, Wenzhou 325035, China.

E-mail: [zoupeng@wmu.edu.cn](mailto:zoupeng@wmu.edu.cn)

* Corresponding author: Daoyong Ni; Address: Affiliated Yueqing Hospital, Wenzhou Medical University, Wenzhou 325035, China.

E-mail: 491837368@qq.com

# These authors contributed equally to this work

Figure S1


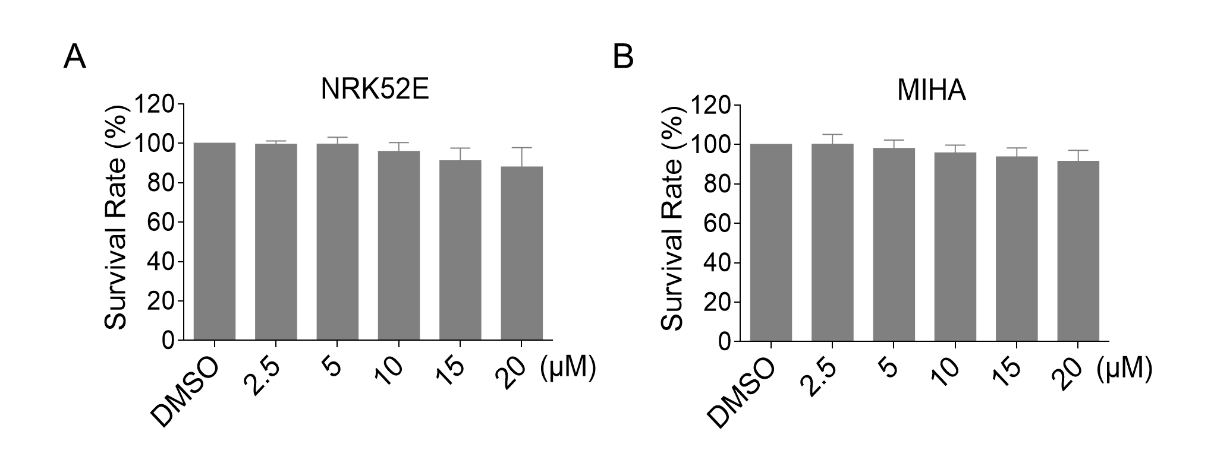


**Figure S1.** **GLB treatment has little effect on normal cells.** (A-B) Cell viability was measured after treated with GLB for 24 h. Data from three technical replicates.
